# Is the Host Viral Response and the Immunogenicity of Vaccines Altered in Pregnancy?

**DOI:** 10.3390/antib9030038

**Published:** 2020-08-04

**Authors:** Zainab Saeed, Orene Greer, Nishel Mohan Shah

**Affiliations:** Academic Department of Obstetrics & Gynaecology, Imperial College London, Level 3, Chelsea & Westminster Hospital, 369 Fulham Road, London SW10 9NH, UK; zainab.saeed14@imperial.ac.uk (Z.S.); o.greer@imperial.ac.uk (O.G.)

**Keywords:** influenza, vaccine, pregnancy, antibodies, B cell

## Abstract

The intricacy of the maternal immune system arises from its ability to prevent a maternal immune response against a semi-allogenic fetus, while protecting the mother against harmful pathogens. However, these immunological adaptations may also make pregnant women vulnerable to developing adverse complications from respiratory viral infections. While the influenza and SARS pandemics support this theory, there is less certainty regarding the clinical impact of SARS-CoV-2 in pregnancy. In the current COVID-19 pandemic, vaccine development is key to public preventative strategies. Whilst most viral vaccines are able to induce a seroprotective antibody response, in some high-risk individuals this may not correlate with clinical protection. Some studies have shown that factors such as age, gender, and chronic illnesses can reduce their effectiveness and in this review, we discuss how pregnancy may affect the efficacy and immunogenicity of vaccines. We present literature to support the hypothesis that pregnant women are more susceptible to respiratory viral infections and may not respond to vaccines as effectively. In particular, we focus on the clinical implications of important respiratory viral infections such as influenza during pregnancy, and the pregnancy induced alterations in important leukocytes such as TFH, cTFH and B cells, which play an important role in generating long-lasting and high-affinity antibodies. Finally, we review how this may affect the efficacy of vaccines against influenza in pregnancy and highlight areas that require further research.

## 1. Introduction

It is interesting to consider whether a pregnancy-specific immune response increases the susceptibility of pregnant women to adverse clinical outcomes in association with respiratory viruses and, if so, what is the nature of this response? Furthermore, pregnancy-specific alterations of the respiratory system may also play a role, contributing to increased morbidity and mortality when compared to that of the general population. The severity of illness in pregnant women observed during recent pandemics have served as important examples of why research in vaccinology in pregnancy is required. The Mothers and Babies: Reducing Risk through Audits and Confidential Enquires across the UK (MBRRACE-UK) report (2009–2012) described the devastating impact of widespread pandemic influenza virus infection in pregnancy, and the subsequent recommendation to introduce an influenza vaccination program in pregnancy was clearly needed [1,2]. Whilst ex vivo seroprotective antibody production post vaccine has been shown in pregnancy, only a moderate reduction in influenza-like illness has been achieved with this vaccination strategy [3,4]. This suggests that pregnancy may be modifying post vaccination responses. In a recent study by our group, we show that whilst post vaccination antibody titres are comparable between pregnant and non-pregnant women, low-level pregnancy related immune regulation leads to an altered post vaccination immune response [4]. Therefore, more research is pivotal in understanding the initial adaptive immune response as well as the memory recall response.

However, inefficient antibody function may not be the cause. Data from the pertussis vaccine, which is given to reduce the incidence of infant pertussis infection through trans-placental antibody transmission, have been shown to be effective. This is both in terms of maternal antibody production and neonatal antibody transfer, and reduced neonatal pertussis infection in those born to vaccinated mothers [5,6,7]. Therefore, the antibodies generated by the mother’s immune system following vaccination are clinically effective in the neonate. Irrespective, clinical data from recent coronavirus infections i.e., Middle East respiratory syndrome-related coronavirus (MERS) and Severe Acute Respiratory Syndrome (SARS) suggest that pregnant women are inherently at a greater risk of respiratory infections, with higher rates of mechanical respiratory support and death [8].

Changes in pregnancy respiratory physiology and anatomy will also negatively affect the clinical outcomes [9]. However, the pregnant lung may also be more susceptible to inflammation and tissue injury [10]. Furthermore, one of the key drivers of clinical severity in these patients is thought to be a heightened cytokine response, which has been reported during the H1N1 pandemic [11]. The cytokine storm, in sepsis, has been shown to cause hypotensive shock by direct effects on the cardiovascular system. For example, cytokines such as Tumor Necrosis Factor-α (TNF-α) and Interleukin-1β (IL-1β) are associated with myocardial depression [12,13]. Furthermore, measurable levels of serum Troponin T, I and B-type natriuretic peptide (BNP) have been shown to be increased with sepsis-associated myocardial depression [14,15,16]. There may be a number of factors that contribute to the severity of illness with respiratory viruses, their downstream effects, and the efficacy of vaccines against them. Physiological changes may influence susceptibility and clinical severity of illness but altered pregnancy-induced immune responses may also play a key role in attenuating antibody responses in pregnancy.

In this perspective piece, we propose the hypothesis that immune responses unique to pregnancy influence the natural history of respiratory viral infections in this population even after vaccination. We present supportive data and highlight areas for further research.

## 2. The Effect of Pregnancy and the Burden of Viral Respiratory Infections

### 2.1. Pregnancy Related Immune Modulation

Pregnancy provides a complex challenge for the maternal immune system as it needs to protect the mother against infections while creating immune tolerance for the paternal antigens and the trophoblast [17,18,19]. The fetus is separated from the mother by a maternal–fetal interface and can be described as semi-allogenic [20]. Medawar proposed that during pregnancy the maternal immune system is systemically suppressed to avoid an immune response against the fetus [21]. This notion, however, has now been replaced by the concept of pregnancy related ”immune modulation” [17,22]. Understanding the mechanisms behind immune modulation in pregnancy holds the key to developing effective treatment plans in pregnancy and more effective vaccines.

The fairly simplistic view that pregnancy can be conceptualised as a single event is an outdated concept [23]. There is increasing evidence to suggest that both cell-mediated and humoral responses vary in different gestational periods and pregnancy can be defined as three separate biological events [23,24]. The first trimester of pregnancy has been described as a ”pro-inflammatory phase” promoting blastocyst implantation and placental development [24]. In fact, implantation within the endometrial tissue, leads to the recruitment of pro-inflammatory Th1 cells and related cytokines, such as IL-1, IL-6, IL-8, leukemia inhibitory factor (LIF), and tumor necrosis factor α (TNF α) [24,25,26]. In contrast, the second trimester has been described as an ”anti-inflammatory” state both systemically and at the maternal–fetal interface with an increase in Th2 related cytokines such as IL-10, IL-4, and TGFβ [21,24]. Parturition is the preparation and the onset of labour and is the third and final stage of pregnancy [21]. It is characterised by the infiltration of neutrophils and macrophages into the myometrium and the upregulation of pro-inflammatory cytokines, such as IL-1β and IL-8, which are associated with the onset of uterine contractions [21,23,24,26].

Tropism towards Th2 or ”anti-inflammatory” responses in pregnancy has been widely studied to explain fetal tolerance [27]. This theory suggests that pregnancy is an anti-inflammatory state induced by producing Th2 related cytokines such as IL-10 and that any shift from this could lead to complications, such as miscarriage, during pregnancy [23]. Several studies looking at unexplained recurrent pregnancy loss in the first trimester reported a decreased frequency of T regulatory cells (Tregs) in circulation and in the decidua when compared to normal pregnancies [28,29]. Tregs, defined as CD4 + CD25 high and expressing FOXP3, have been shown to have regulatory properties when the expression of CD25 is maintained and are necessary to resolve the pro-inflammatory effects of the immune system [30,31]. It has also been shown that T-helper 17 (Th17) cells increased in women who suffered unexplained recurrent miscarriages, while Tregs were lower [32]. During labour, the immune suppressive effects of Tregs are targeted towards Th2 subsets, thus appearing to support inflammation [33,34]. This indicates the delicate balance which is needed to maintain a successful pregnancy while also protecting the fetus.

Understanding the role of key antigen presenting cells (APCs), such as dendritic cells (DCs) is important when discussing cell-mediated and humoral immunity in pregnancy. In particular, the total number of DCs as well as their subsets are altered at different gestational stages when compared to the non-pregnant state [19]. Two major DC subsets include myeloid dendritic cells (mDCs) that migrate to the tissues and plasmacytoid (pDCs) that mainly reside in the peripheral blood and have anti-viral functions [19,35]. A study by Bella et al. found that the absolute number of DCs was higher during the first trimester when compared to non-pregnant women, but it progressively decreased with each trimester [35]. Moreover, the authors also demonstrated a decline in pDCs as the pregnancy progressed, and this is similar to another study which showed a lower percentage of pDCs in pregnant women primarily in the third trimester [35,36]. Interestingly, both studies also showed an increase in activated pDCs during the third trimester, which is parallel with the notion of increased inflammation in the third trimester [19,35,36]. Using allogenic mice models, it has been shown that the production of Type 1 interferons (IFN) is reduced in pregnant mice following H1N1 infection, and high levels of progesterone (P4) may contribute to the decreased activation of DCs [37,38]. Vanders et al. also demonstrated that in pregnancy there was an increased expression of an inhibitory ligand PDL1 in pDCs following pH1N1/09 infection, which may suppress T cell mediated immunity [36]. While this may be necessary for fetal tolerance, the increase in PDL1 could also account for dampened anti-viral maternal T cell responses and the increased morbidity in pregnancy following influenza infection. DCs are also crucial in building a bridge between the innate and adaptive immune system as they can phagocytose pathogens and induce T cell activation and differentiation into effector subsets [39]. This, in turn, is important for antibody class switching, affinity maturation, and inducing an effective humoral response [39].

The role of P4 in the immune system has been of interest especially in pregnancy, and very little is known about its effect on the adaptive immune system. One study hypothesised that this may be due to P4′s ability to block thymide in lymphocytes resulting in decreased cell-mediated immunity [40]. There is a 5–10-fold increase in serum P4 in pregnancy and up to a 50–100-fold increase in the maternal–fetal interface tissues [41]. High concentrations of P4 in the maternal tissue have been shown to have immune modulating effects on local immune cells [40,42]. Interestingly, a study by Lissauer et al. characterised the effect of P4 at 10 µM concentration, similar to that seen at the maternal–fetal interface, and found that this altered the functional properties of both CD4 and CD8 T cells in pregnancy [43]. There was also a marked reduction in IFN-γ and TNF-α by both CD4 and CD8 T cells, and an increase in Th2 associated cytokine IL-4, particularly by CD8+ T cells [43]. At lower concentrations of 1 µM, which is similar to levels in the maternal serum, the effect on peripheral immune cells may be more subtle [42,43]. P4 has also been shown to favour FOXP3+ Treg differentiation of cord blood T cells and suppress Th17 differentiation [44]. It should, however, be noted that the authors did not find the same effect in adult PBMCs. P4 receptor positive T lymphocytes can synthesise progesterone-induced blocking factor (PIBF), which can skew a shift to Th2 type from Th1 in the periphery [45]. The immunological effect of P4 is complex and more work is needed to fully understand its effects on the peripheral adaptive immune system and the mechanisms underpinning that effect. P4 related immune modulation in pregnancy may be more pronounced in the maternal–fetal interface; however, we cannot rule out any subtle effects on the peripheral immune cells [43,46].

### 2.2. Is Pregnancy Associated with a Modified Clinical Response to Respiratory Virus 

Particular infections have been associated with a greater severity of illness in pregnancy; influenza is infamous for its differential effect. The morbidity and mortality associated with the disease has disproportionately affected women in pregnancy [47]. This has been notable with successive viral pandemics (H1N1 between 1918–1919 and 2009 and also H2N2 in 1957–1958) [48,49,50,51]. The 1918–1919 pandemic was associated with a 27% case fatality rate, and in 2009, 6% of influenza infected ICU patients that died were pregnant [52]. This is striking when you consider that pregnant women constitute 1% of the population globally.

During an acute respiratory viral illness there are probably a number of factors that contribute to the severity of illness. It is understood that physiological changes of pregnancy can facilitate entry of the pathogen. For example, adaptations in the vaginal mucosal architecture in pregnancy, associated with increased vascularity, have been implicated in the increased risk of ascending uterine infection and/or transmission of pathogens to the circulation. A similar mechanism may be at work in the respiratory epithelium of the nasopharyngeal cavity which is hyperaemic and oedematous in pregnancy in response to elevated levels of circulating P4 [9]. With respect to the influenza vaccine, while serum IgG is an established correlate of seroprotection, nasal IgA is emerging as another useful measure of protection from influenza, as well as vaccine efficacy [53]. This may be more relevant in pregnancy where susceptibility to respiratory infection and subsequent complications are greater than in non-pregnant women [54].

Although hormonal influences predominate, advancing pregnancy leads to anatomical changes, which may have a slight impact on respiration due to elevation of the diaphragm by the gravid uterus. Residual volume and functional residual capacity are reduced; predisposing to a rapid decrease in oxygenation in association with airway inflammation [55]. Sepsis can be associated with increased pulmonary microvascular pressures and capillary permeability and the development of acute respiratory distress syndrome (ARDS) [56]. Additionally, in line with that of the systemic vasculature (due to nitric oxide and P4 reducing vascular tone), there is a reduction in pulmonary vascular resistance and as plasma colloid pressure is reduced there is an increased risk in patients “tipping” into pulmonary oedema [56,57]. Bhatia et al. have suggested that interstitial lung tissue may have a higher fluid content in pregnancy [55]. P4 has been implicated in increased water retention within the lung tissue, reducing the lung’s diffusion capacity, and potentially exacerbating hypoxaemia in an already compromised airway [58]. Difficulties in mechanically ventilating patients in pregnancy compound these issues in the critically ill.

A large amount has been documented concerning the clinical impact in pregnancy of influenza. It is interesting to consider whether other respiratory viruses such as SARS, MERS, and SARS-CoV-2 are also particularly virulent in pregnancy and, if so, why? What is known, is that in pregnancy, the risk of developing subsequent respiratory complications following infection with viral pneumonia are far greater when compared to the general population. It is possible that these outcomes are likely to be made worse by the compressive effect of the gravid uterus on the maternal lungs and a greater chance of basal atelectasis during late pregnancy [9]. SARS has been associated with a disproportionate clinical impact on pregnant women and a recent literature search reported a case fatality rate of 15%, and a comparative analysis of morbidity and mortality of SARS positive pregnant and non-pregnant women reported both higher ICU admission rates and mortality rates. The same study reported that for MERS infection, 64% of pregnant women were admitted to the ICU, and the case fatality rate was reported to be as high as 27%. It is these data that alerted the global community to the potential risks in pregnancy of the novel coronavirus SARS-CoV-2. There may be a number of factors that contribute to this. While physiological changes of pregnancy may facilitate entry of the pathogen, an altered pregnancy-induced immune response may play a key role in the modulation of the normally well-coordinated antibody response to the initial adaptive response phase as well as to the recall response, which in pregnancy, might be pivotal.

It is likely to be too early to determine accurate case fatality rates for the ongoing SARS-CoV-2 pandemic [59]. Estimates, initially, have been as high as 3%–5%. Critical cases are predominantly complicated by severe respiratory failure and the systemic effects of the cytokine storm lead to multi-organ failure [60]. Pregnancy-specific adaptations of the immune response may impact the host response, altering the natural history of infection. In pregnancy, patients do not have a greater morbidity and mortality than their non-pregnant counterparts. Patients have been predominantly affected in the third trimester. While a great number of infected patients have been asymptomatic or suffered mild–moderate symptoms, admissions to ICU have been documented, as well as the escalation of treatment for extracorporeal membranous oxygenation (ECMO) [59].

What we know from the data thus far surrounding COVID-19 infection is that males, patients over 70, Black, Asian, or minority ethnic origin (BAME) patients, those with obesity, and those with comorbidities are at the greatest risk of ICU admission and death following infection with SARS-CoV-2. However, at the outset of the SARS-CoV-2 pandemic, pregnant women were placed in the high-risk category with respect to the expectations of the severity of illness that they would experience if they contracted the virus. This public health measure was extrapolated from our knowledge of influenza in pregnancy. As events have unfolded, the current consensus is that there may not be an increased risk of adverse maternal clinical outcome although our view, in agreement with others, is that the available data, to date, are limited [61,62]. The consensus, thus far, is based predominantly on published case series and it would be expected that a more definitive assessment can be made following analysis of the results of large population studies from around the world.

Of note, Knight et al., performed a prospective cohort study in the UK of 427 women and reported the incidence of hospital admissions for confirmed SARS-CoV-2 as 4.9 in 1000 maternities (95% CI 4.5–5.4) [63]. This study used data obtained from the UK Obstetric Surveillance System (UKOSS), which is a national reporting system in the UK. In line with the trends seen in the general population, specific sub-groups of pregnant women were at increased risk of admission; these included advanced maternal age (aOR 1.35, 95% CI 1.01–1.81), those with comorbidities (aOR 1.52, 95% CI 1.12–2.06), and BAME women (aOR 4.49, 95% CI 3.37–6.00), which were the highest risk pregnancy group. The UKOSS data also showed that most hospitalised women were in the second half of their pregnancy. In these patients, 40 women (9%) required critical care, 4 women (1%) required extracorporeal membranous oxygenation (ECMO) and there were 5 fatalities (1%). While the majority of women were well and discharged home, at completion of the study, there were 30 (7%) remaining inpatients. Consistent with studies performed in other populations, fetal and neonatal transmission was uncommon and neonatal admissions in the 427 women were <1.5%. Despite this, there has been an association of adverse pregnancy outcomes with maternal infection [64].

The UKOSS reporting appears to suggest that hospitalisation in the pregnant population is uncommon. It is difficult to determine what role early public health interventions to protect pregnant women may have had on these findings. Given that the majority of adverse clinical outcomes occurred in patients that were older, males and/ or BAME, we believe that an improved understanding of the role of pregnancy in the host response to this infection may be aided by studies comparing the rates of level 3 critical care admission and death in pregnancy with that of demographically matched non-pregnant females. If pregnant patients are more susceptible than their non-pregnant counterparts to developing COVID-19, what are the proposed mechanisms of SARS-CoV-2 virulence in pregnancy.

### 2.3. ACE-R and Pregnancy

Highly expressed in type II alveolar epithelial cells (as well as arterial/ venous endothelium and arterial smooth muscle cells), the angiotensin converting enzyme-2 (ACE-2) receptor has been identified as the gateway to infection for the SARS and SARS-CoV-2 coronaviruses, with SARS-CoV-2 having a 10-fold increased affinity than its predecessor [65,66,67]. The binding of the SARS-CoV-2 spike (S) protein to ACE-2 receptors initiates the fusion of the viral membrane (composed of a lipid bilayer and transmembranous proteins) with the host alveolar cell membrane and facilitates the passage of the viral RNA which then hijacks the host mechanisms of protein synthesis for replication [68]. Li et al. used RNA-Seq to determine the tissue expression of ACE-2 receptors across a number of human tissue compartments [69]. While the data did not show any differences in ACE-2 receptor expression with gender, age, or ethnicity, correlations between ACE-2 and immune signatures in the lungs showed a positive correlation in older patients. In addition, Smith et al. looked specifically at ACE-2 expression in the lung in smokers and reported an upregulation of ACE-2 receptors in these patients [70]. However, while ACE-R expression is important, variation within the amino-acid sequence of the ACE-2 receptor binding domain is associated with differing binding affinity, and receptor polymorphisms may correlate with clinical severity of infection [66]. Therefore, evaluating these features may be more relevant in determining the risk of viral entry. Furthermore, SARS-CoV-2 infection leads to the up-regulation of ACE-2 receptors in the lung [71]. Using a method of gene expression interpretation called Gene Set Enrichment Analysis (GSEA), the same group propose that ACE-2 may mediate post-infection immune regulation via innate and adaptive responses [71]. More specifically, they investigated the ACE-2 expression in SARS-CoV-2 infected epithelial cells and found elevated ACE-2 expression and cytokine secretion (IL-1, IL-10, and IL-6) in association with B and T cell activation and an increase in T-cell cytokine secretion [71]. Additionally, there was an increase in viral cell entry and transcription [71].

Research is required in pregnant women to determine how ACE-2 receptor expression in the respiratory tract compares to non-pregnant individuals, and whether this contributes to an increased susceptibility to severe illness with the coronaviruses. In any event, as discussed, this may not play a significant role in the natural history of the infection. While there does not appear to be differential viral transmission among adults, males, the elderly, and BAME groups, patients appear to be disproportionately affected by infection. Thus, although tissue expression in pregnancy may be a moot point (but an important evaluation), analysis of pregnancy-specific variations of the receptor binding domain amino-acid sequence and its affinity for the SARS-CoV-2 S protein would be of value.

## 3. What Is the Current Knowledge on Antibody Producing B Cells and Where is More Research Required?

Antibody secreting cells (ASCs), which comprise plasma cells or memory B cells, arise from activated B cells that have previously entered the germinal centre (GC) [72]. ASCs are vital for the production of effective antibodies and are terminally differentiated plasma cells or plasmablasts [73]. GC B cell maturation and antibody production is very much dependent on a specialised group of helper T cells [74]. T follicular helper cells (T_FH_) are a specialised subset of effector CD4 T cells that induce B cell activation, and differentiation into class-switched and antibody secreting long-lasting plasmablasts [75,76,77,78,79]. This support provided by T_FH_ cells is vital for an effective humoral response during natural infection and/or following vaccination [80,81]. In humans, T_FH_ cells are currently defined by their high expression of CXCR5, inducible co-stimulator (ICOS), and Programmed cell death protein-1 (PD-1 and CD279) [81,82]. However, there is also a subset of circulatory T_FH_ (cT_FH_) that express ICOS, PD-1, and CCR7, and these markers have been used to phenotype subtypes of cT_FH_ [82,83]. One noticeable difference observed between tissue T_FH_ and cT_FH_ cells is that cT_FH_ have a lower expression of ICOS and PD-1, and do not express Bcl-6. Furthermore, they produce an increased amount of interleukin-2 (IL-2), which makes them an important subset for B cell activation [80]. ICOS in particular has been shown to consistently reflect cT_FH_ activation after parenteral vaccination [84]. Furthermore, it has been reported that CXCR3^+^ T_FH_1 like cells increase after influenza vaccination with the expression of ICOS and PD-1 also increasing [75]. This has been shown to strongly correlate with an increase in the avidity of antibodies secreted from the memory B cell pool by potential selection of high affinity B cells at extrafollicular sites [75,85]. Whilst our knowledge of T_FH_ cells is rapidly improving, very little is still known about their function in the circulation and how these subsets compare to T_FH_ cells in the germinal centres [77].

To date, only one study has looked at the frequency of circulatory T_FH_ (cT_FH_) and their activation in pregnancy. This study showed that the frequency of CD45RO^+^ CXCR5^+^ expressing cT_FH_ cells, which are thought to represent the circulatory counterpart to GC T_FH_ with similar functions, are increased in pregnancy and these cT_FH_ also express more ICOS when compared with non-pregnant controls [86,87]. No difference was observed with PD-1 expression, but this study concluded that pregnancy favours an expanded functional cT_FH_ subset, which will favour an enhanced humoral response. This is particularly useful, as it would suggest that in spite of a pro-inflammatory biased response to infection during pregnancy, the maternal immune system still has the capacity to respond to vaccinations and produce functional high affinity antibodies. Interestingly, ageing studies have also found that the frequency of cT_FH_ cells are increased in the elderly when compared to younger groups [88,89]. However, these cells display a lower level of activation [88,89]. There have been contrasting findings in other studies. For example, one study showed a 35% decrease in cT_FH_ cells in an elderly cohort, but these cells also showed an increase in ICOS expression [75,79,85]. The authors hypothesised that the increased ICOS expression may be due to non-specific inflammation, and in fact the reduced proportions of cT_FH_ cells was in keeping with a less efficient antibody response.

The humoral response in pregnancy is a delicate balance between protecting the fetus and the mother, as an influx of auto-antibodies, such as antiphospholipid antibodies (aPL), can lead to severe complications including preeclampsia and intrauterine fetal death [90,91]. Having said that, Taylor and Hancock in the 1970s demonstrated that, in the absence of maternal IgG, cultured trophoblasts were vulnerable to cell-mediated cytotoxicity [90].The anti-paternal IgG may provide protection for the growing fetus. Our knowledge of B cell subsets and the generation of antibodies in natural infection and/or through vaccination is still deficient in pregnancy and more research is required in this area. There has, however, been some work on the regulatory B cell subset (Bregs). This subset of B cells can suppress inflammatory responses, in particular responses during autoimmune disease and organ transplant [92]. Lima et al., showed that Bregs, defined as CD24^high^ CD38^high^, increased during the first and third trimester when compared to non-pregnant women [91]. This may be necessary to dampen the T cell mediated responses, which could result in the rejection of the fetus [91]. Bregs proportions also appeared to peak in the postnatal period, and these cells may function to suppress the pro-inflammatory state following labour and during the recovery phase. In contrast, the frequency of other B cell subsets decreased during the third trimester, suggesting an attenuated B cell response during late pregnancy [91]. Similarly, in both ageing and HIV-1 infection, the number of B cells have been shown to decrease, and this has been attributed to the reduced expression of transcriptional factors, such as E12 and E47, which influence B cell maturation [93]. Additionally, it has also been observed that the number of GCs is reduced during ageing and this in turn leads to a reduction in high-affinity antibodies [94]. In pregnancy, there has been a suggestion that the number of GCs is decreased alongside thymic atrophy [95]. Together, these data suggest that antibody responses in pregnancy are enhanced in some respects, but some features may also be suppressed. Clearly more research is needed in antibody and B cell function in pregnancy.

## 4. Benefits of Maternal Immunisation and the Immunogenicity of Vaccines in Pregnancy? 

Vaccination during pregnancy can not only protect the mother against harmful pathogens, but also protect the neonate after birth [96]. Immunisations during pregnancy are usually classified into three groups: routinely administered, administered based on medical need when there is significant benefit to the mother or fetus, or avoided due to potential risks [97]. Major types of commercially available vaccines include live attenuated, inactivated, toxoid, and subunit, recombinant and conjugate vaccines [98]. Live attenuated vaccines contain an altered or weakened live pathogen, which mimics exposure to natural infections and generates long-lasting immunity [98]. In theory, live attenuated vaccines can also revert to virulent wild-type strains and so these vaccines are not usually administered to either immunocompromised individuals or pregnant women due to the risk of severe infection and perinatal infections, respectively [98,99]. Inactivated and toxoid vaccines are manufactured by inactivating or killing the pathogen, and the immune response to these vaccines is often less potent than live attenuated vaccines and titres of newly produced antibodies in response to the vaccine tend to decline over time and boosters are usually required [98]. Subunit vaccines are composed of at least one antigen or fragments from the pathogen of interest, which can invoke a sufficient immune response [98,99]. Finally, conjugate vaccines rely on the same principle as recombinant vaccines, but they combine pieces of bacterial coat with a carrier protein. The carrier protein is essential in mounting an adaptive immune response to generate long term immunity [98].

### 4.1. Routinely Administered Vaccines in Pregnancy

#### 4.1.1. Tetanus, Diphtheria, and Acellular Pertussis (Tdap)

Rates of infant pertussis-related complications (pulmonary, neurological, and nutritional) and even infant death have persisted in developed countries with rates increasing in recent years [97,100]. Pertussis vaccines are licensed for use after 6 weeks of age; however, most vaccines are not administered till at least 8 weeks of life [100]. A study conducted in the US highlighted that 64% of all pertussis-related infant deaths during 1991–2008 occurred in infants younger than 6 weeks old, and therefore, newborns are at most risk [101]. The first infant dose may only induce partial immunity and therefore additional measures of protection are required [101]. Alternative strategies to control the disease in infants includes ”cocooning” the neonates for an appropriate period of time and maternal immunisation [102]. Maternal immunisation of pertussis was originally recommended within a narrow window in the third trimester, ideally between 27 and 36 weeks of gestation, and this has now been adopted by more than 20 countries including the US and the UK [100,102]. Protective antibodies have been shown to transfer from the mother to the fetus trans-placentally and this can provide protection to infants too young for a primary immunisation course [103]. However, the optimal timeframe for transferring the antibodies is vital as the half-life of pertussis-specific antibodies is relatively short [100]. A study by Ebenhardt et al. compared maternal Tdap immunisation during the second trimester (GW 13–25) vs. the third trimester (≥GW 26) and found that the increase in geometric mean concentrations (GMC) of cord blood antibodies to recombinant pertussis toxin (PT) was greater in the second trimester when compared to the third. The authors proposed recommending Tdap from the second trimester to increase uptake of the vaccine [100]. Since these data were published, UK recommendations now suggest vaccination between 16 and 32 weeks of gestation as this has been shown to maximise neonatal seroprotection and provide protection to babies born preterm [100]. Safety of any vaccination during pregnancy is of the utmost concern. Several studies have shown that Tdap was safe to administer during pregnancy and was not associated with preterm or still births following the vaccination [104,105].

#### 4.1.2. Influenza Vaccine 

The World Health Organisation (WHO) characterises the following groups of people as high risk: elderly, pregnant women, children under 5 years, individuals with chronic medical and immunosuppressive conditions, and healthcare workers [106]. During the 2009 H1N1 pandemic, pregnant women were seven times more likely to suffer severe influenza-associated complications and twice as likely to die than non-pregnant women [38]. Furthermore, one study showed that of all women hospitalised during the first wave of the H1N1 pandemic in California, 42.6% were either pregnant or postpartum. A total of 22% of these patients required intensive care and 8% died [107,108]. Our understanding of the underlying mechanisms behind why pregnant women have increased mortality and mobility following influenza infections remains unclear [38]. Studies have reported that following influenza infection, pregnant mice showed higher viral loads, reduced body weight, and increased damage to the lungs when compared to non-pregnant mice [109]. Increased infiltration of pulmonary neutrophils and macrophages may contribute to tissue injury in their lungs resulting in an increased mortality rate in pregnant mice [110]. Therefore, WHO recommends that all pregnant women should receive the seasonal influenza vaccine.

Vaccination programs are the most effective method of preventing the community spread of influenza and it has been shown that influenza vaccines, in particular, reduce the risk of influenza-like illness in both mothers and neonates [111,112]. A study by Blanchard-Rohner et al. demonstrated that 84%–86% of newborns of vaccinated women showed seroprotective levels depending on the strain [113]. Additionally, they showed that mothers who were vaccinated against influenza at least two weeks prior to delivery had 5–17 times higher geometric mean titres in the umbilical cord blood depending on the strain and interval between vaccination and delivery [102,113]. However, in vaccination studies, the incidence of influenza-like illness is still greater in pregnancy when compared to non-pregnant women [3]. There is limited research available on the immunogenicity and efficacy of influenza vaccination in pregnant women when compared to non-pregnant individuals. Currently, there are 26 licensed inactivated vaccines for influenza with trivalent inactivated virus (TIV) being the most frequently distributed. However, new WHO recommendations are shifting towards quadrivalent vaccines (QIV) [111,114]. TIV vaccines are composed of three strains, which include two strains from the influenza A subtype (H1N1 and H3N2), and one from the influenza B subtype [111]. QIV contains an additional strain from influenza B subtype, which commonly includes B/Yamagata or B/Victoria [111]. Inactivated influenza vaccines (IVs) are produced by propagation of the influenza virus in hens′ eggs and the virus is inactivated using formaldehyde or b-propiolactone [115]. The vaccine is available in three main manufacturing formulations: whole virus, split (chemically disrupted), and subunit (purified HA and NA proteins) [114,115]. The intranasal live attenuated influenza vaccine (LAIV) is rarely administered to pregnant women as any associated risks to the mother or fetus are largely unknown [116]. A study examining 834,999 pregnancies in the US found that only 138 pregnant women (0.017%) were given the LAIV and any incidences reported following the vaccination were at a similar rate to unvaccinated pregnant women [116]. Therefore, while it may be best to avoid LAIV in pregnancy, it may not cause harm if used for medical reasons.

Influenza vaccines primarily work by inducing a protective immune response against the HA glycoprotein, and the vaccines are licensed on over seventy percent of individuals achieving a HAI titre >1:40 or a 4-fold increase in over forty percent of individuals [111,114]. A vaccines’ ability to induce an antigen-specific HAI titre may not be a correlate for protection in the community, and studies have shown that factors such as age, gender, chronic infections, and illnesses can reduce the effectiveness of vaccines for clinical protection [117]. Seroprotection may not always translate into clinical protection for populations with poor immune responses. Additionally, the degree of protection after vaccination is also dependent on matching the antigenic strains to the ones circulating correctly [111].

A recent study carried out by Tregoning et al. investigated the effect of pregnancy on the transcriptomic response after vaccination in pregnant and non-pregnant mouse models and from humans. They also found no difference between the pregnant and non-pregnant state at the level of individual genes, and found similar levels of inflammation post vaccination in both mice models and humans [118]. Kay et al., also demonstrated that pregnant women were able to induce a robust plasmablast response following vaccination as these women had an equivalent rate of post vaccination HAI titres and seroprotection when compared to non-pregnant women [119]. It should, however, be noted that this study had a limited number of samples, which may have reduced the statistical power of the study. In people over 65, the vaccine efficacy against influenza A virus is predicted to be around 20%, and this may be linked to increased inflammation as a result of immunosenescence [120]. The increase in inflammatory markers during the first and third trimester may resemble some features of immunosenescence, but we do not have enough data to support the hypothesis that pregnancy alone reduces the effectiveness of influenza vaccine responses. Pregnant women are at a greater risk for developing complications from respiratory viral infections, and more research is needed in determining whether the efficacy and immunogenicity of vaccination in pregnancy are attenuated.

### 4.2. Vaccines Available during Pregnancy if Medically Necessary

The following vaccines may be given to pregnant women if medical staff deem them necessary to protect the mother and the fetus: Hepatitis B vaccine, Hepatitis A vaccine, pneumococcal vaccine, meningococcal vaccine, poliomyelitis vaccine, Japanese encephalitis vaccine, yellow fever vaccine, typhoid fever vaccine, anthrax vaccine, and smallpox vaccine [97]. Hepatitis A is transmitted through the faecal–oral route via contaminated food or drink or close contact with an infected person [102]. Hepatitis A vaccine is an inactivated vaccine and is classified as low risk and given if clinically necessary [102]. In the case of yellow fever, if pregnant women are travelling to, or live in, endemic areas, a live attenuated vaccine may be given as the risk of the vaccine outweighs the risk of infection to the mother and fetus [121]. The immunogenicity and efficacy of these vaccines is largely unknown and needs to be investigated further.

### 4.3. Vaccines Avoided during Pregnancy 

Live attenuated vaccines have the potential to cross the placental barrier and cause infection to the fetus, thus their administration is contraindicated during pregnancy [97]. The Measles, Mumps, and Rubella (MMR) vaccine is often given to children and clinical staff are advised against giving it to women who are either planning on getting pregnant or are already pregnant [97,98]. The potential risk to the fetus is theoretical; however, the consequences of severe infection during pregnancy outweigh the benefit of the vaccine [98].

### 4.4. Maternal Vaccines being Developed

RSV infects almost 100% of children by 2 years of age and is a major cause of lower respiratory tract illness in infants from 6 weeks to 6 months [122]. Risk factors associated with increased morbidity and mortality include preterm infants, underlying chronic lung or heart disease, and immunogenicity [123]. Reinfections of RSV are common as immunological memory to the virus is low, thus the development of a vaccine is imperative to create long term immunity. In order to reduce the disease burden in infants, maternal immunisation is a plausible strategy to boost serum neutralising antibody responses during the second and third trimester that could be transferred to the fetus through the placenta [122]. A recombinant RSV vaccine is currently in phase II clinical trials in non-pregnant women with plans of extending research in pregnant women and entering phase I trials [124]. High concentrations of maternal antibodies after maternal immunisation are essential for the maximum transfer of maternal antibodies to the fetus [125]. Therefore, the question of whether vaccine immunogenicity is altered in pregnancy is an important one and needs to be studied further in a wider range of vaccines.

## 5. Summary

In this perspective, we show that pregnancy is a unique state where physiological and immunological adaptations may increase the risk of severe infection. Important leukocytes such as T_FH_, cT_FH_, and B cells, that are important for antibody production, may behave differently in pregnancy. Despite this, antibody titres post influenza vaccination are comparable between pregnant and non-pregnant women. However, the influenza and coronavirus pandemics have highlighted the vulnerability of pregnant women to developing serious complications from these infections. Whilst pregnant women, alongside other risk groups, have been at the forefront of vaccination efforts due to their high-risk status, there is emerging evidence that vaccine efficacy might be negatively influenced by pregnancy. New methods of measuring vaccine efficacy in high risk groups, including pregnancy, may help to determine if these responses are indeed altered. In the long term, research focused on pregnancy immune responses to vaccines is certainly required.
